# Human platelet lysate produced from leukoreduction filter contents enables sufficient MSC growth

**DOI:** 10.1186/s13287-025-04329-y

**Published:** 2025-04-23

**Authors:** Shinobu Wakamoto, Tomoko Furukawa, Masahito Kawabori, Mitsuaki Akino, Shiho Kato, Hisae Fuse, Sumio Ohtsuki, Yoshihiro Torimoto, Miki Fujimura, Shuichi Kino

**Affiliations:** 1 Japanese Red Cross Hokkaido Block Blood Center, Sapporo, Japan; 2 R&D Division, RAINBOW. INC, Sapporo, Japan; 3 Department of Neurosurgery, Faculty of Medicine, Hokkaido University, Sapporo, Japan; 4 Department of Pharmaceutical Microbiology, Faculty of Life Sciences, Kumamoto University, Kumamoto, Japan; 5 Japanese Red Cross Blood Service Headquarters, Tokyo, Japan

**Keywords:** Platelet lysate, Mesenchymal stem cell, Leukoreduction filter, Platelet, Plasma

## Abstract

**Background:**

Stem cell therapy holds significant potential for promoting recovery, with numerous products currently under development. Blood-derived supplements are often essential for successful stem cell expansion, with fetal bovine serum (FBS) being the most commonly used supplement. However, FBS has drawbacks, including the risk of immune responses, ethical concerns about animal welfare, and potential zoonotic infections. Human platelet lysate (hPL), derived from lysed platelets, contains various growth factors and has been proposed as an alternative to FBS. However, obtaining sufficient human platelets for clinical use remains challenging. Leukoreduction filters, used during blood transfusion manufacturing to remove leukocytes, also retain significant amounts of platelets and plasma. This study investigates the feasibility and efficacy of filter-derived hPL (f-hPL) for mesenchymal stem cell (MSC) expansion.

**Methods:**

Leukoreduction filters were collected after their use in the manufacturing of whole blood transfusion products. Each filter was reverse-perfused with saline to extract residual blood contents. Platelets (f-platelet) and supernatant were separated by multiple centrifugation steps. f-Platelet were lysed with varying concentrations of fresh frozen plasma (FFP) to determine the optimal protein concentration for the lysate solution. Then, plasma left in the leukoreduction filters were used to generate lysate solution (f-plasma) at optimal protein concentration. f-Platelet (1.1 × 10^9^/mL) and f-plasma (27 mg/mL protein) were combined in a freezing bag and subjected to three freeze-thaw cycles to produce f-hPL. Both small- and large-scale f-hPL were manufactured, and MSCs expansion and quality assessments were perfomed to evaluate the efficacy of f-hPL.

**Results:**

A total of 3.5 *±* 0.6 × 10^10^ f-platelets were obtained from a single leukoreduction filter, yielding a collection efficiency of 37.1 ± 5.3%. The optimal protein concentration of lysate solution for cell expansion was > 27 mg/mL. Subsequently, six leukoreduction filters used to produce enough f-platelet and p-plasma for 100 mL of f-hPL. MSCs cultured in medium supplemented with 10% f-hPL demonstrated superior expansion, with cell proliferation rates 20% higher than those observed with commercial hPL and 300% higher than those cultured with FBS. The expanded MSCs met the International Society for Cell & Gene Therapy criteria for cell surface markers and differentiation potential. MSCs expanded with f-hPL expressed similar to or higher amounts of hepatocyte growth factor compared with those cultured with FBS and human AB serum. Furthermore, f-hPL significantly enhanced cell proliferation up to P12 and effectively prevented cell senescence.

**Conclusion:**

f-hPL derived from leukoreduction filters demonstrated strong capacity for MSCs expansion. The use of discarded blood materials for regenerative medicine represents a sustainable and efficient approach, with significant therapeutic potential.

**Supplementary Information:**

The online version contains supplementary material available at 10.1186/s13287-025-04329-y.

## Introduction

Cell transplantation therapy holds promise for promoting recovery in various diseases. To date, several cell types have been investigated, including embryonic stem cells, induced pluripotent stem cells, and mesenchymal stem cells (MSC). In most cases, blood-derived supplements are required to be added in the culture medium to successfully expand stem cells, with fetal bovine serum (FBS) being the most commonly used supplement [1]. However, FBS poses potential risks, including zoonotic contamination, immunological reactions to xenogeneic serum antigens, and ethical concerns regarding animal welfare [2–4]. An alternative candidate is human AB serum, which offers the advantage of non-zoonotic contamination, and many clinical trials are currently utilizing human AB serum for cell expansion [5, 6]. However, due to its reliance on human resources, the cost of this supplement is relatively high, and its supply may be insufficient to meet the growing demand for industrial-scale stem cell products. Recent reports have demonstrated that human platelet lysate (hPL) is a promising substitute for cell supplement [7, 8], and several clinical trials have incorporated hPL as a supplement in stem cell culture media [9–15]. Similar to human AB serum, the preparation of a sufficient quantity of hPL is crucial for clinical applications. However, human platelets are primarily collected at blood banks for transfusion purposes, rather than for hPL production. Consequently, outdated platelet concentrates, which account for 2–4% of the total platelet concentrates, are frequently utilized for hPL production. Due to ongoing efforts to minimize outdated blood products, the availability of such platelet concentrates is expected to decrease, potentially limiting their use as a supplement for cell growth [16, 17]. 

Leukoreduction filters are widely employed to remove leukocytes from whole blood during the production of blood transfusion products, thereby preventing immunological reactions due to donor leukocytes. Currently, these filters are discarded as biomedical waste after the filtering process. Interestingly, these filters not only capture leukocytes but also retain approximately 30–99% of the platelets present in whole blood. We hypothesized that these filters could serve as an alternative source of platelets and plasma for the production of platelet lysate. Therefore, in this study, we aimed to establish the optimal procedures for producing hPL from leukoreduction filters (f-hPL) and to evaluate the efficacy of f-hPL.

## Methods

Experimental protocols were approved by the Ethics Committee of the Hokkaido University Hospital (approval number: 012–0334), and Ethic Committee of the Red Cross Society (approved number: 2022-019-3). Written agreement is obtained at the time of blood donation from each volunteer for the use of blood sample for experimental use, and at the time of bone marrow harvest. The MSCs obtained from Lonza comply with the ethical approval for the acquisition of human tissue for research cell products (further details can be found online at https://www.lonza.com/specialized-modalities/cell-and-gene/tissue-acquisition).

### Preparing leukoreduction filters

**Fig. 1:**
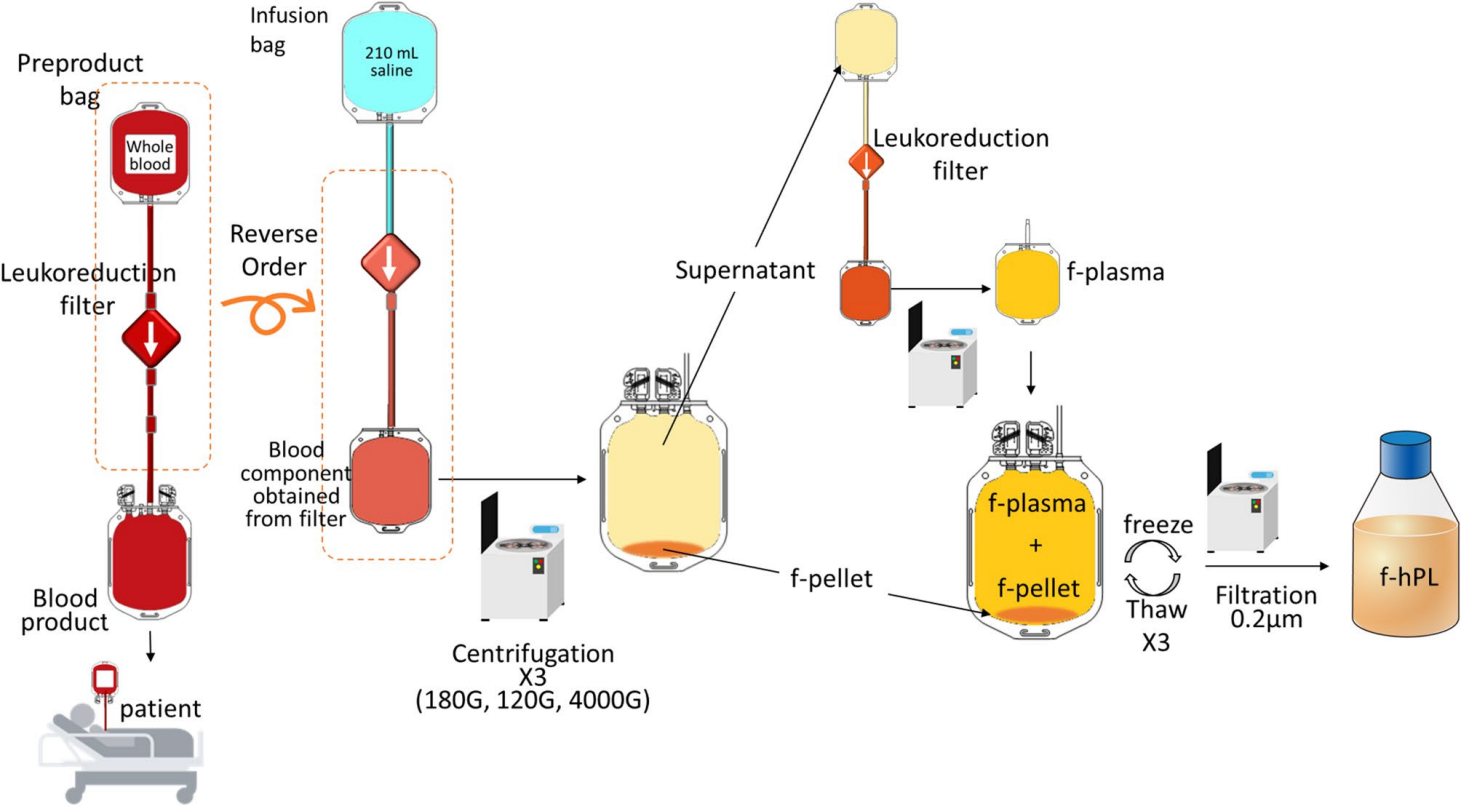
Schematic Representation of f-hPL Production The leukoreduction filter is reversely perfused with saline to recover the residual contents within the filter. The collected contents are then subjected to centrifugation to obtain the f-pellet and supernatant. The supernatant is subsequently passed through an additional leukoreduction filter to achieve a sufficient protein concentration (27 mg/mL) for f-plasma. f-platelet and f-plasma are then combined, followed by three freeze-thaw cycles to facilitate platelet membrane disruption. After purification through centrifugation and filtration, the final f-hPL is collected

Blood was collected by voluntary, non-remunerated donations from healthy individuals at Japanese Red Cross Hokkaido Block Blood Center, Sapporo. A detailed procedure is illustrated in Fig. 1. After obtaining 400 mL of whole blood from the volunteer in the pre-product bag, it was dripped through leukoreduction filter (Sepacell RZ-2000 N, Asahi Kasei Medical, Tokyo, Japan) into the blood transfusion product bag. Subsequently, the product bag was disconnected for subsequent use for blood product. The filter connected to the pre-product bag was maintained at 4 °C until the following day, during which time testing for infectious diseases (syphilis, hepatitis B virus, hepatitis C virus, human immunodeficiency virus, human T-cell lymphotropic virus type 1, and human T-cell lymphotropic virus type 1) was performed and confirmed to be negative. Detailed procedures to prevent bacterial and viral contamination are outlined in the supplementary methods.

### f-platelet collection from leukoreduction filter

Distal end of the filter was aseptically connected with infusion bag (Hicaliq IVH bag, HC-B1006A, Terumo, Tokyo, Japan) using TSCD-II connector (Terumo, Tokyo, Japan). Each Hicaliq bag contained 210 mL of saline inside, then, the contents trapped inside the leukoreduction filter were collected into the pre-product bag by dripping 210mL of saline in reverse order, then pre-product bag is disconnected with filter and Hicaliq bag. Two ABO identical pre-product bags were gathered to obtain approximately 420 mL of blood contents sample. Then, the bag was centrifuged (180G, 10 min, room temperature; RT, 9942, KUBOTA, Tokyo, Japan), after which the supernatant was transferred to a new blood bag (KBP-66DC, SB-Kawasumi, Tokyo, Japan) and centrifuged again (120G, 10 min, RT) to remove red blood cell and leukocyte. After the second centrifugation procedure, the supernatant was collected into the blood bag (KBP-66DC, SB-Kawasumi, Tokyo, Japan). A small amount of sample was taken from the blood bag to evaluate the number of platelets, residual red blood cell and leukocyte (Automated blood cell counter XN-1000 with the Blood Bank mode, Sysmex, Hyogo, Japan). Subsequently, the bag was centrifuged (4000G, 20 min, RT, 9920, KUBOTA, Tokyo, Japan) to obtain platelet pellet (filter-platelet; f-platelet), and supernatant, which are then cryopreserved in the − 80 °C until subsequent use.

### Protein concentration of platelet lysate solution

To determine the optimal protein concentrations for the preparation of platelet lysate solutions, f-platelets were dissolved in a filtered supernatant containing varying concentrations of expired-FFP. The platelet concentration was set to 1.1 × 10^9^/mL. The protein concentrations of the supernatant and expired-FFP were 5.8 mg/mL and 65 mg/mL, respectively. By incorporating 0%, 20%, 35%, 40%, 70%, and 90% expired-FFP into the filtered supernatant, platelet lysate solutions were generated with protein concentrations of 6, 18, 27, 30, 48, and 60 mg/mL, respectively. These concentrations were evaluated using the BCA protein assay kit (23225, Thermo Fisher Scientific). f-platelet dissolved in solutions with varying protein concentrations underwent three freeze-thaw cycles (-80 °C for more than 4.5 h followed by 4 °C for more than 20 h) to ensure complete disruption of the platelet membrane. Platelet lysates were subsequently collected via centrifugation (4000 g, 20 min, 4 °C) and filtration (0.2 μm, Thermo Fisher Scientific). To identify the optimal protein concentration for cell expansion, 10% platelet lysate at different protein concentrations was added to MEM-α (Thermo Fisher Scientific), along with heparin (2 IU/mL) and gentamicin (40 µg/mL). Bone marrow-derived MSCs (Lonza, Walkersville, MD, USA) were seeded at 5 × 10^3^ cells per cm^2^ in a 24-well plate containing the aforementioned medium. FFP without f-platelet, commercially available hPL (nLivenPL^TX^, PL-PR-100, Sexton Biotechnologies, IN, US) and fetal bovine serum (Equitech-Bio Inc., TX, US, Gibco, 12662-029, NY, US) served as controls. The cells were harvested and counted (Luna-FL, Logos Biosystems, South Korea) five days after seeding, and the fold increase in cell number was calculated as the ratio of the final cell count to the number of initially seeded cells. Microscopic images were captured to assess cell morphology.

### Preparation of filtered-human platelet lysate (f-hPL)

In the previous experiment, a protein concentration exceeding 27 mg/mL was determined to be essential for optimal cell expansion. The filtered supernatant was subsequently passed through the filter again to achieve a protein concentration of 27 mg/mL (f-plasma). Thereafter, f-platelets (1.1 × 10^9^/mL) were added to the f-plasma (27 mg/mL) to generate f-hPL (Fig. 1).

Small-scale production of f-hPL was initially conducted, yielding 226 mL of f-hPL from 15 filters. Cell expansion was evaluated using MSCs under the previously described conditions using 24-well. 10% of small-scale f-hPL, hPL, and FBS were incorporated into the medium to compare the impact on cell growth and cell surface marker.

Subsequently, large-scale production of f-hPL was carried out using 284–292 filters to obtain 4.7 L of f-hPL. The product was pooled in 5 L bag (STD FLAXAFE 5 L) (5 L/lot), and filtration process was performed using biowelder TC (Sartorius Stedim Biotech), filter (Sartopore 2 XLG 0.2 μm), a peristaltic pump (630 S/R; WatsonMarlow). Cell expansion was further evaluated using MSCs as previously described. 10% of f-hPL or FBS were incorporated into the medium to compare the impact on cell growth for large-scale f-hPL.

### Evaluation of MSC supplemented with f-hPL

Large-scale f-hPL is used for subsequent analysis. 10% of f-hPL, FBS, or human AB serum (H6910, Sigma-Aldrich, MO, USA) were added to MEM-α as previously described, to compare cell quantity and quality between the different supplements. Three different lots of MSCs (Passage 4; P4) were seeded into flasks using the aforementioned medium and incubated for 5 days. The cells were then trypsinized, washed, and counted, after which they were re-seeded into 24-well plates with MEM-α without supplements. Cell supernatants were collected on day 3, and were analyzed for the expression of hepatocyte growth factor (HGF) using a Human HGF ELISA kit (DHG00B, R&D Systems, Minneapolis, MN, USA), and subjected to proteomic analysis. HGF expression was normalized to the cell number on day 3, as MSC expansion varied between the pre-exposed supplements. The supernatant was digested with trypsin and analyzed by mass spectrometry for proteomic profiling as previously described [18, 19]. Detailed procedures of proteomics are provided in the supplementary method.

Additionally, cell senescence analysis was performed. Previously described MSCs were continuously cultured in 12-well plates up to P12 with different supplements. Cells were harvested and counted every 4 days. Cumulative population doubling (PD) was calculated using the following formula: PD = [log10(Nh)– log10(Ni)] / log10 [2], where Nh represents the cell number at the time of harvest, and Ni is the cell number at the time of seeding. β-galactosidase (β-gal) expression was analyzed for cells with different supplements at P5, P8, and P12. Cells were stained using the Cellular Senescence Detection Kit-SPiDER-βGal (Dojindo, Tokyo, Japan). Fluorescence intensity was measured from three randomly selected, non-overlapping regions of interest (ROIs) by using an automated fluorescence counter application (BZ-X Analyzer, Keyence Co., Osaka, Japan).

### Evaluation of large-scale MSC production using f-hPL

The Automated Cell Expansion System (Quantum cell expansion system, Terumo BCT, CO, USA) was employed to assess the cell expansion capacity for clinically relevant scale. 10% f-hPL was added to the previously described medium components (MEM-a, heparin, and gentamicin) to produce 10 L of medium. Fifty milliliters of freshly extracted bone marrow were loaded onto the system according to the manufacturer’s instructions. Continuous medium flow (0.1–4.0 mL/min) was maintained to feed the MSCs, and daily lactate concentrations were monitored to estimate the number of proliferating cells. Cells were harvested from the system 21 days after bone marrow input, and cell number, viability, size, surface markers and differentiation capacities were evaluated as previously reported [10]. Antibodies and kits used to assess surface antigens and differentiation capacities are listed in the supplementary table.

### Statistics

All assessments were conducted by blinded investigators. Data are expressed as the mean *±* standard deviation (SD). Statistical analyses were performed using JMP Pro 14 software (SAS Institute Inc., NC, USA), or GraphPad Prism version 9.0.1, (Graphpad Software, MA, USA). Statistical comparisons between two different groups were made using the t-test or Wilcoxon test, the differences between three or more groups were examined using a one-factor analysis of variance (ANOVA) followed by Turkey’s HSD test. Probability values of *p* < 0.05 were considered statistically significant.

## Results

### f-platelet and supernatant solution from leukoreduction filter

A total of 596 filters were evaluated for subsequent analysis. The average number of platelets collected from a single filter was 3.5 *±* 0.6 × 10^10^, yielding a collection efficiency of 37.1 ± 5.3%. The levels of white blood cell and red blood cell contamination from each filter were 4.1 *±* 4.0 × 10^4^ cells and 11.3 *±* 4.5 × 10^6^ cells, respectively.

### Optimal protein concentration for platelet lysate solution

**Fig. 2:**
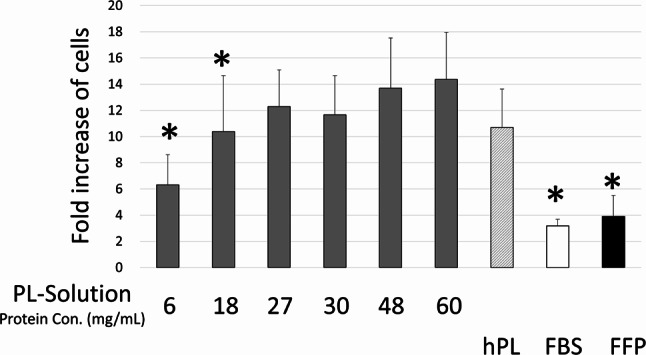
Impact of protein concentration of lysate solution on MSC proliferation. MSC were grown with platelet lysate containing different protein concentration of lysate solution (6, 18, 27, 30, 48, and 60 mg/mL). The results of the cell expansion assay revealed that protein concentrations ranging from 27 to 60 mg/mL did not exhibit statistically significant differences. However, notable differences were observed between the 60 mg/mL group and the 6 mg/mL, 18 mg/mL, FBS, and FFP groups. Results are expressed as mean ± SD of MSCs from three donors. *p < 0.05 compared to f-hPL plasma protein of 60 mg/mL

**Fig. 3:**
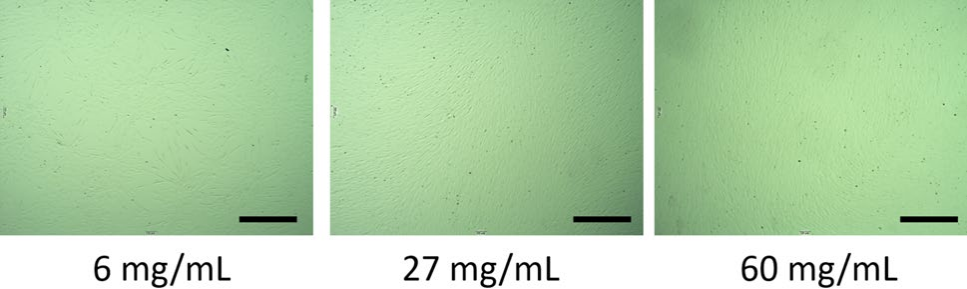
Microscopic cell morphology. MSCs were expanded using human platelet lysate with varying protein concentrations in the lysate solution. The 27 mg/mL and 60 mg/mL groups exhibited the characteristic spindle-shaped morphology of MSCs, whereas the 6 mg/mL group displayed an irregular and non-uniform cell shape. Bar = 500 μm

Given that the leukoreduction filter is perfused with saline, the plasma component is considered significantly lower than that in outdated platelet concentrates, which may influence the quality of f-hPL. Various protein concentrations in the platelet lysate solution were evaluated for optimal cell expansion. The results of the cell expansion assay revealed that higher protein concentrations in the platelet lysate solution facilitated superior cell expansion (Fig. 2). Protein concentrations ranging from 27 to 60 mg/mL did not exhibit statistically significant differences in cell expansion. However, notable differences were observed between the 60 mg/mL group and the 6 mg/mL, 18 mg/mL, FBS, and FFP groups. The fold increase in cell number for the 60 mg/mL group was nearly four times greater than that of the FBS group. These findings indicate that the protein concentration in the platelet lysate solution is crucial for enhanced cell expansion. Interestingly, the suboptimal cell expansion observed in the FFP group, which contains sufficient protein but lacks f-platelets, suggests that both the platelet content and the solution protein are critical for effective platelet lysate preparation. Microscopic analysis further revealed abnormal cell morphology in the 6 mg/mL group, while normal spindle-shaped morphology was observed in the 27 and 60 mg/mL groups (Fig. 3). Based on these results, a platelet lysate solution with a protein concentration of 27 mg/mL was selected for subsequent analyses.

### MSC expansion quality of small-scale and large-scale f-hPL

**Fig. 4:**
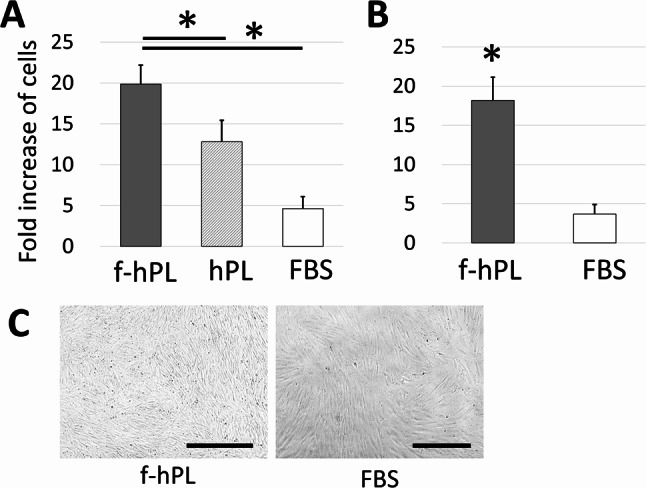
Cell expansion potential for small and large-scale production of f-hPL. (**A**) The MSCs expansion capacity was assessed using both small-scale (approximately 50 filters) and large-scale (approximately 150 filters) production of f-hPL. Small-scale f-hPL demonstrated significantly higher cell expansion potential compared to commercial platelet lysate (PL) and fetal bovine serum (FBS). (**B**) Similarly, large-scale f-hPL exhibited significantly greater cell expansion capacity compared to FBS. (**C**) Microscopic analysis of MSCs expanded with large-scale f-hPL and FBS revealed the characteristic spindle-shaped morphology of MSCs. Bar = 500 μm

The quality of f-hPL under small (approx. 15 filters) and large (approx. 290 filters) scale productions were evaluated. Both products successfully expand the MSC four times more cells compared with that with commercially available hPL or FBS (Fig. 4A: small scale, Fig. 4B large scale). Microscopic analysis of MSCs expanded with large-scale f-hPL and FBS revealed the characteristic spindle-shaped morphology of MSCs (Fig. 4C). The surface markers of cell were further evaluated with large scale f-hPL, which the expanded cells met the criteria of the International Society for Cell & Gene Therapy (ISCT) criteria for cell surface markers (Table 1).

**Table 1:** Surface marker expression of MSCs grown in the medium supplemented with large scale production of f-hPL

Surface Marker	f-hPL #1	f-hPL #2	f-hPL #3
CD90	99.8%	99.9%	99.9%
CD73	95.4%	99.2%	98.6%
CD105	97.2%	98.1%	95.9%
CD44	99.6%	99.7%	99.5%
CD166	96.6%	98.3%	94.6%
CD34	0.0%	0.1%	0.1%
CD45	1.4%	0.6%	1.1%
CD19	0.1%	0.1%	0.1%
CD14	0.1%	0.1%	0.0%
HLA-DR	0.6%	1.1%	0.9%

### MSC performance with f-hPL

**Fig. 5:**
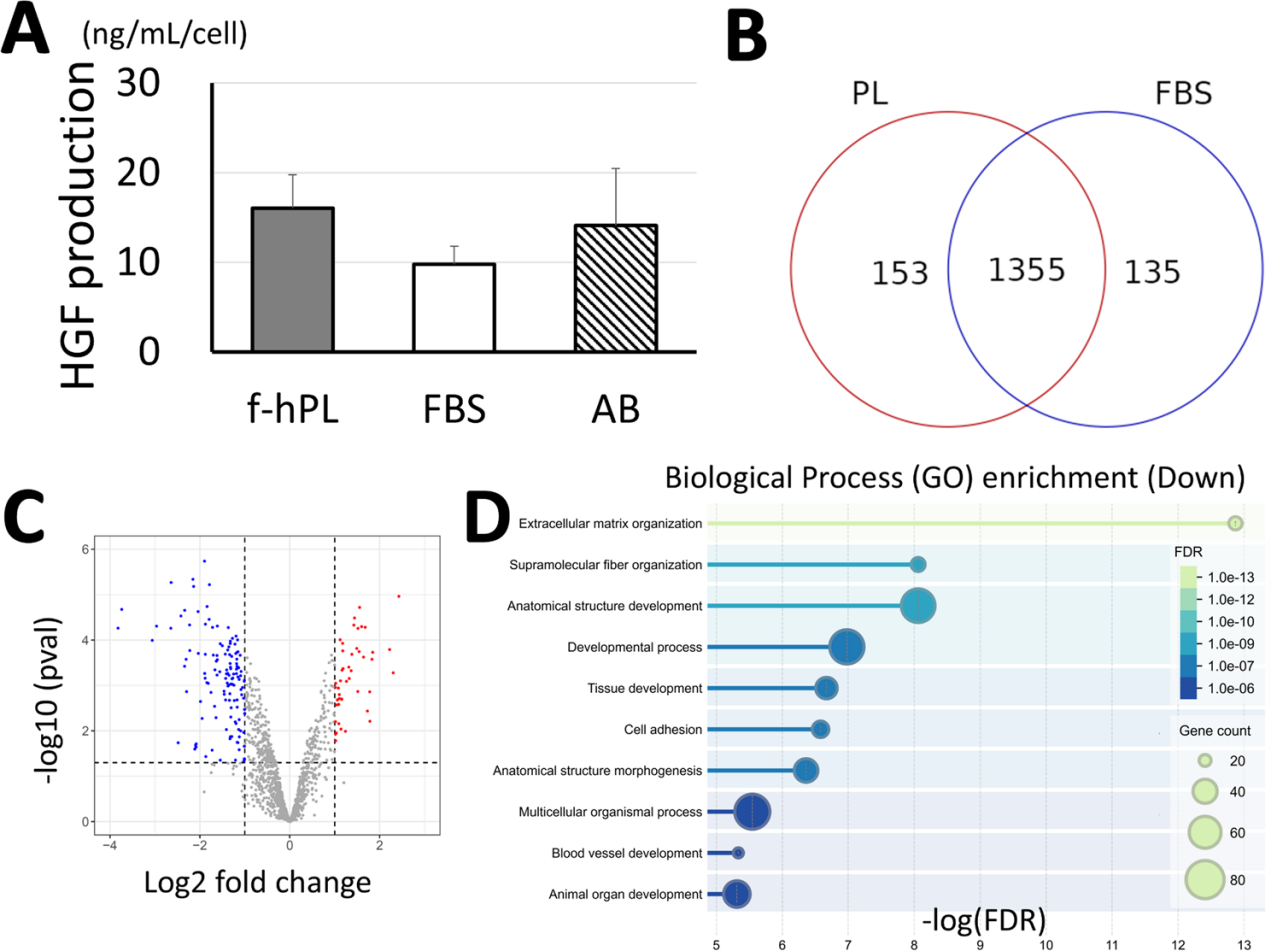
MSC expression profile with different supplements. (**A**) Hepatocyte growth factor expression profile of MSC supernatant treated with f-hPL, FBS, or human AB serum. f-hPL demonstrated similar to superior expression compared with other supplements. (*n* = 3 each). (**B**) Venn diagram of proteomics analysis showed that proteins released from cells pre-exposed to f-hPL displayed a predominantly similar expression profile compared to those exposed to FBS. While differential expression of proteins was observed between the groups, with 153 proteins exhibiting higher expression and 135 proteins showing lower expression in the f-hPL group. (**C**) Volcano plots of the identified protein. (**D**) Biological process Gene Ontology (GO) enrichment analysis revealed that extracellular matrix organization was downregulated in the f-hPL group. *n* = 3 each

Cell performance with respect to the different supplements added to the culture media was evaluated. HGF expression analysis revealed that MSCs expanded with f-hPL exhibited a similar to superior expression profile when compared to those cultured with FBS and human AB serum (Fig. 5A). Proteomic analysis of the cell supernatants showed that proteins released from cells pre-exposed to f-hPL displayed a predominantly similar expression profile compared to those exposed to FBS, with 1,355 proteins (82%) showing similar expression levels (Fig. 5B, C). However, differential expression of proteins was observed between the groups, with 153 proteins exhibiting higher expression and 135 proteins showing lower expression. Biological process Gene Ontology (GO) enrichment analysis revealed that RNA splicing and mRNA processing were upregulated, while extracellular matrix organization was downregulated in the f-hPL group, suggesting active cell proliferation (Fig. 5D, Supple Fig. 1A). Protein degree analysis further indicated that MSCs treated with f-hPL exhibited significantly higher expression of stemness-related proteins (Myc targets v1), while showing reduced expression of collagen matrix proteins, suggesting enhanced cell mobility for better cell expansion (Supplementary Fig. 1B, C). Data comparing f-hPL to human AB serum were consistent with the findings observed between f-hPL and FBS (data not shown).

**Fig. 6:**
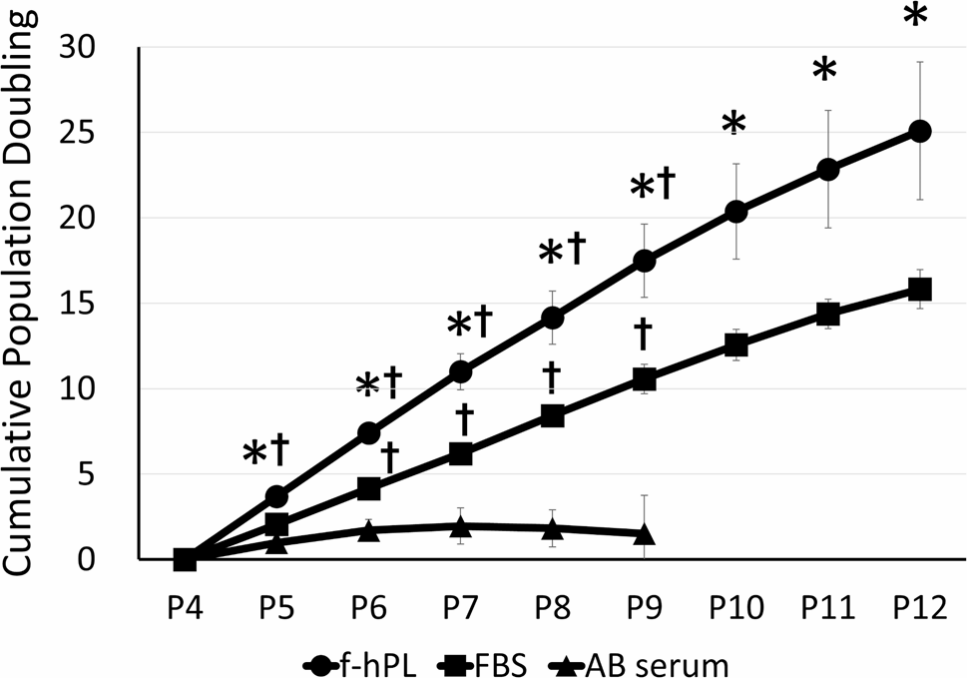
Cumulative cell expansion with different supplements f-hPL facilitated significantly better MSCl expansion compared to both FBS and human AB serum up to P12. AB serum group culture were ceased after P9 because of the low cell division. *n* = 3 each *p < 0.05 compared to FBS, ^**†**^*p* < 0.05 compared to human AB serum

**Fig. 7:**
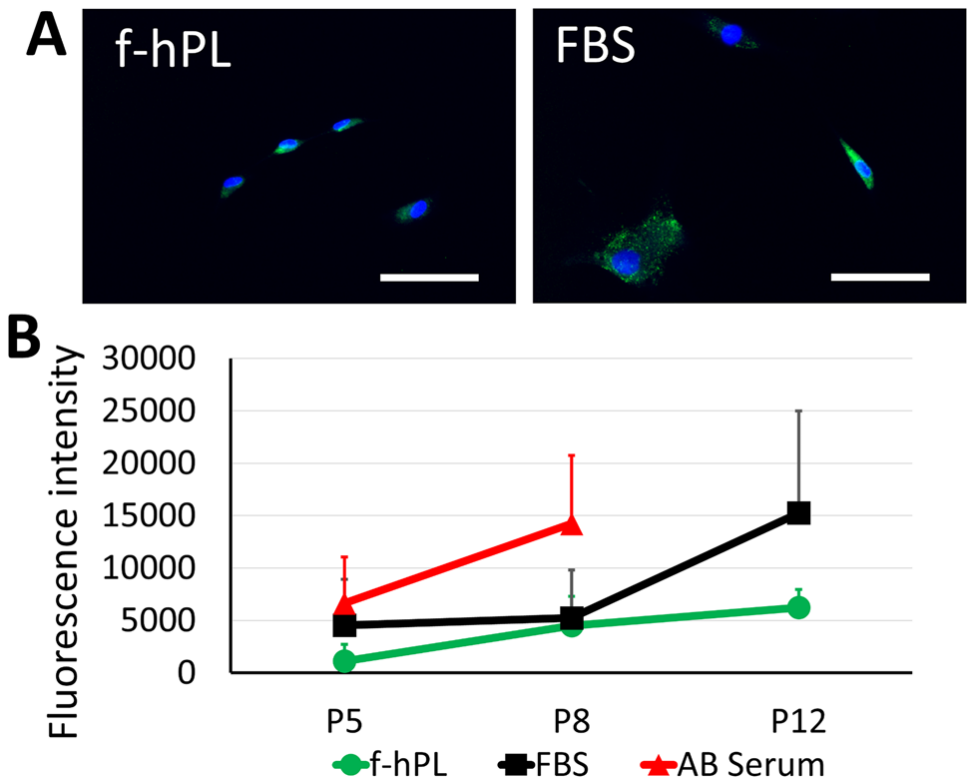
Cell senescence analysis between different supplements. (**A**) β-gal staining of the MSC treated with either f-hPL or FBS after P12. Significantly high signals are seen in the FBS group. (**B**) Fluorescence intensity of MSC treated with different supplements were compared regarding different passages. human AB serum exhibited a higher trend of β-gal expression as early as P8, whereas FBS and f-hPL showed lower levels of expression. MSCs treated with FBS exhibited substantial β-gal expression at P12, whereas f-hPL-treated MSCs displayed comparatively lower expression. *n* = 3 each, Bar = 100 μm

Cell senescence was also evaluated across the different supplements. f-hPL facilitated significantly better cell expansion compared to both FBS and human AB serum up to P12 (Fig. 6). β-gal expression analysis further confirmed that human AB serum exhibited a higher trend of β-gal expression as early as P8, whereas FBS and f-hPL showed lower levels of expression. MSCs treated with FBS exhibited substantial β-gal expression at P12, whereas f-hPL-treated MSCs displayed comparatively lower expression (Fig. 7A, B). Considering the superior population doubling observed in the f-hPL group, which suggests that these cells underwent more divisions, the reduced senescence observed at the same passage indicates a better anti-senescence profile in the f-hPL group.

### Clinical scale cell expansion using f-hPL

**Fig. 8:**
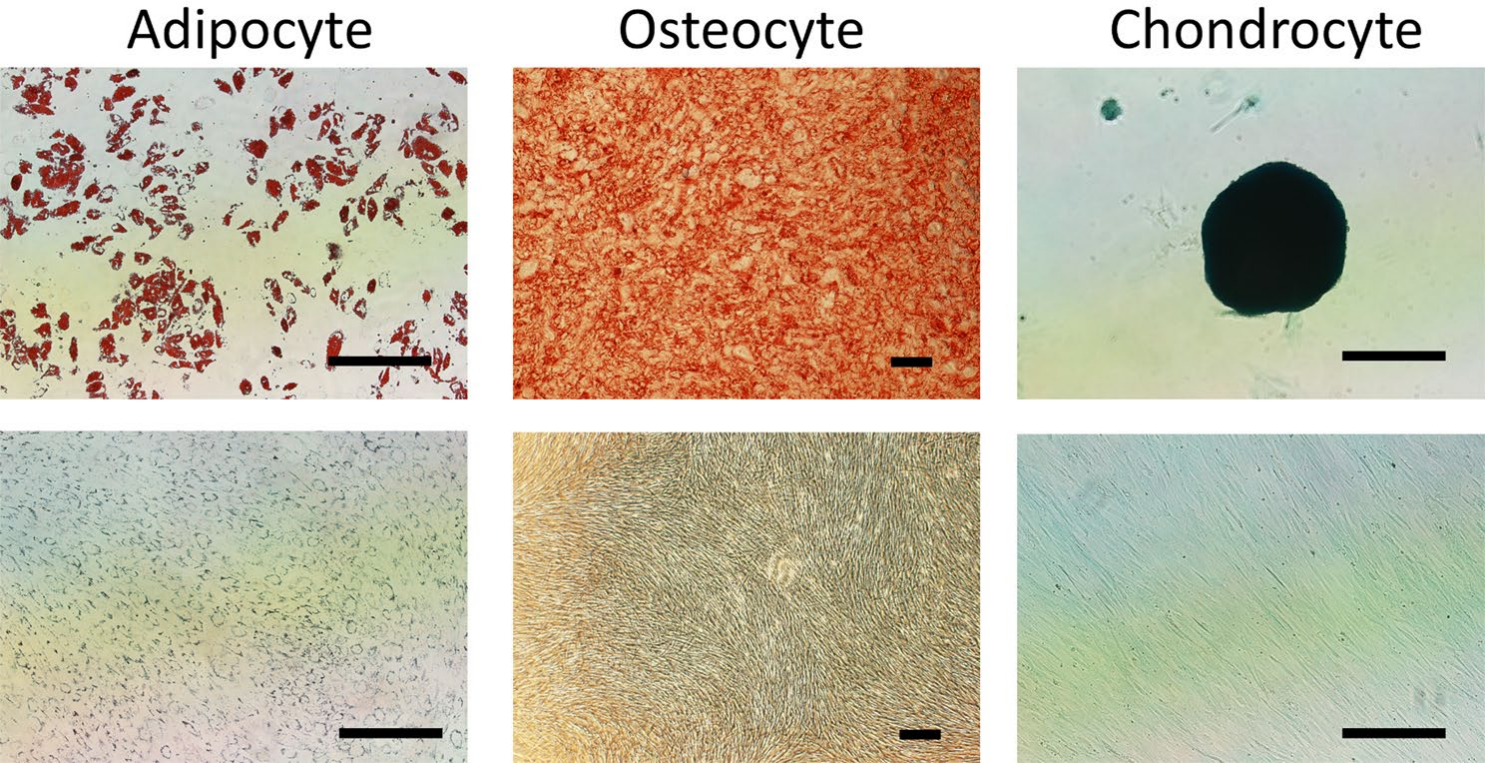
MSC differentiation potential. MSCs expanded with large-scale f-hPL and cultured in the Quantum system demonstrated robust differentiation potential into three distinct lineages: adipogenesis, osteogenesis, and chondrogenesis. (Lower raw: control) Bar = 200 μm

The f-hPL was further tested using an automated cell culture system. Three different bone marrow samples were obtained from volunteers and loaded onto the Quantum Cell Expansion System. Platelet-derived growth factor-BB (PDGF-BB) levels were monitored to assess the quality of the medium, with concentrations ranging from 12.4 to 17.7 ng/mL. MSCs were successfully expanded using the f-hPL-containing medium, with cell numbers ranging from 4.6 to 12.6 × 10^7^ cells, demonstrating high viability (> 90%). The cells exhibited appropriate surface markers and differentiation potential (Table 2; Fig. 8).

**Table 2:** Surface marker expression of MSCs grown in the medium supplemented with large scale production of f-hPL in quantum system

		BM 1	BM2	BM3
f-hPL	PDGF-BB (ng/mL)	12.4	17.7	10.5
Automated cell culture	Quantity of Bone marrow (mL)	50	50	50
	Age of donor (Y)	23	25	22
	Total cell number	4.6 × 10^7^	12.6 × 10^7^	8.4 × 10^7^
	Viability	91.8%	95.3%	89.4%
	Cell size (um)	16.5	21.5	15.6
FACS	Isotype control	2.0%	1.7%	0.9%
	CD45	3.7%	1.9%	2.3%
	CD44	94.5%	99.5%	99.7%
	CD90	95.3%	99.5%	99.5%
	CD105	92.9%	96.6%	91.7%
	CD166	88.8%	93.0%	82.1%

## Discussion

In the present study, we demonstrate that human platelet lysate (hPL) can be successfully generated using residual components from the leukoreduction filter. The cell expansion capacity of filtered hPL (f-hPL) was comparable to that of commercial platelet lysate (PL) and superior to that of fetal bovine serum (FBS). Additionally, we identified that a protein concentration greater than 27 mg/mL in the plasma is a key factor in the generation of f-hPL. The f-hPL showed similar to superior functional profile of MSC compared with FBS and human AB serum.

Numerous preparation methods for platelet concentrates have been explored, including the buffy coat method, platelet-rich plasma method, and single-donor apheresis [20]. These methods have been shown to efficiently produce hPL for MSC expansion. However, the use of these platelet concentrates competes with standard clinical treatments for patient care. In contrast, our study utilized material that would otherwise be discarded in the process, presenting a novel approach that aligns with the concept of responsible consumption and production as outlined in the United Nations’ Sustainable Development Goals (SDGs). We found that a protein concentration exceeding 27 mg/mL is suitable for f-plasma, which is approximately half the concentration of platelet concentrates approved for use in Japan (67 mg/mL) [21, 22]. Furthermore, we discovered that neither platelet nor plasma alone is sufficient to achieve optimal cell expansion; rather, their combination results in a synergistic effect that enhances cell growth. It is known that approximately 60% of the protein content in plasma is albumin, followed by 15% immunoglobulin G and 3% fibrinogen [23, 24]. however, the specific factors contributing to this synergistic effect warrant further investigation.

Platelet-derived growth factor-BB (PDGF-BB) in hPL is considered a critical factor for MSC expansion, as we previously reported a linear correlation between PDGF-BB levels in the medium and the extent of MSC expansion [25]. The PDGF-BB concentration in f-hPL ranged from 12.4 to 17.7 ng/mL, which is nearly double that of previously reported hPL derived from a similar platelet number (20 units), which yielded 5–6 ng/mL of PDGF-BB. This difference is likely due to the distinct procedural process employed in our study, wherein complement inactivation (56 °C for 30 min) was not performed. While heat inactivation is traditionally used to reduce immune responses from residual complement in blood products, it has also been reported to decrease supplemental factors in FBS [26]. Whether complement inactivation is necessary for MSC production remains uncertain.

In this experiment, we utilized filters that met the same viral and bacterial criteria established for blood products. This approach minimizes the risk of contamination to levels comparable to those of standard blood transfusion products. However, as with blood products, the window period for viral contamination cannot be entirely eliminated. Gamma irradiation to inactivate viral agents is necessary for future human applications.

We compared f-hPL with FBS and human AB serum in terms of both cell expansion capacity and MSC quality. It has been reported that MSCs become larger and more flattened as senescence progresses [27], with an increase in β-gal expression through activation of the p53/p21 and p16 pathways [28]. Our results demonstrate superior cell expansion with f-hPL up to P12, which correlates with a lower senescence profile. These findings are consistent with previous reports that platelet lysate (PL) downregulates apoptotic and senescence-related genes in MSCs, while avoiding tumor-associated genetic alterations [29]. In proteomic analysis, extracellular matrix organization was downregulated, though the precise mechanisms underlying this phenomenon remain unclear. Based on our observations, MSCs treated with f-hPL exhibited improved detachment properties compared to those treated with FBS, suggesting that changes in MSC morphology related to extracellular matrix organization may contribute to this difference.

Several limitations in our study warrant further analysis. First, we were unable to identify the specific proteins in the supernatant that contribute to the enhanced proliferation capacity of f-hPL. This is particularly important for the development of synthetic cell expansion supplements that do not rely on human-derived components. However, due to the complex array of platelet functions, fully elucidating the candidate proteins or exosomes remains a challenge. Second, the optimal platelet concentration for f-hPL was not assessed in this study. While we hypothesize that a higher platelet concentration leads to greater cell proliferation capacity, determining the balance between efficacy and economic feasibility is crucial for the commercial distribution of hPL. Third, we were unable to evaluate other leukoreduction filters, including platelet-sparing filters, as they are not utilized in Japan for blood preparation. Nevertheless, our findings, which suggest that a protein concentration of greater than 27 mg/mL is optimal for supernatant plasma, can serve as an important benchmark for preparing hPL from these filters.

## Conclusion

f-hPL derived from leukoreduction filters exhibited a robust capacity for MSC expansion. The utilization of discarded blood components for regenerative medicine offers a sustainable and efficient approach with considerable therapeutic potential.

## Electronic supplementary material

Below is the link to the electronic supplementary material.

Supplementary Material 1: Supplementary table. Kit used for evaluating cell quality

Supplementary Material 2: Supplementary Figure. (A) Biological process Gene Ontology (GO) enrichment analysis revealed that RNA binding was significantly upregulated in the f-hPL group. (B) Protein degree analysis revealed that stemness marker (Myc targets v1) were upregulated in f-hPL group, while epithelial mesenchymal transition was downregulated. (C) Protein-protein interaction network analysis of downregulated proteins.

Supplementary Material 3

Supplementary Material 4

## Data Availability

The data is currently not deposited in the public repository. The data that support the findings of this study are available from the corresponding author, [MK], upon reasonable request.
