# Supplementary figures and images for: Human platelet lysate produced from leukoreduction filter contents enables sufficient MSC growth
Source: Stem Cell Res Ther. 2025 Apr 23;16:205. doi: 10.1186/s13287-025-04329-y (PMC12020118; doi:10.1186/s13287-025-04329-y)

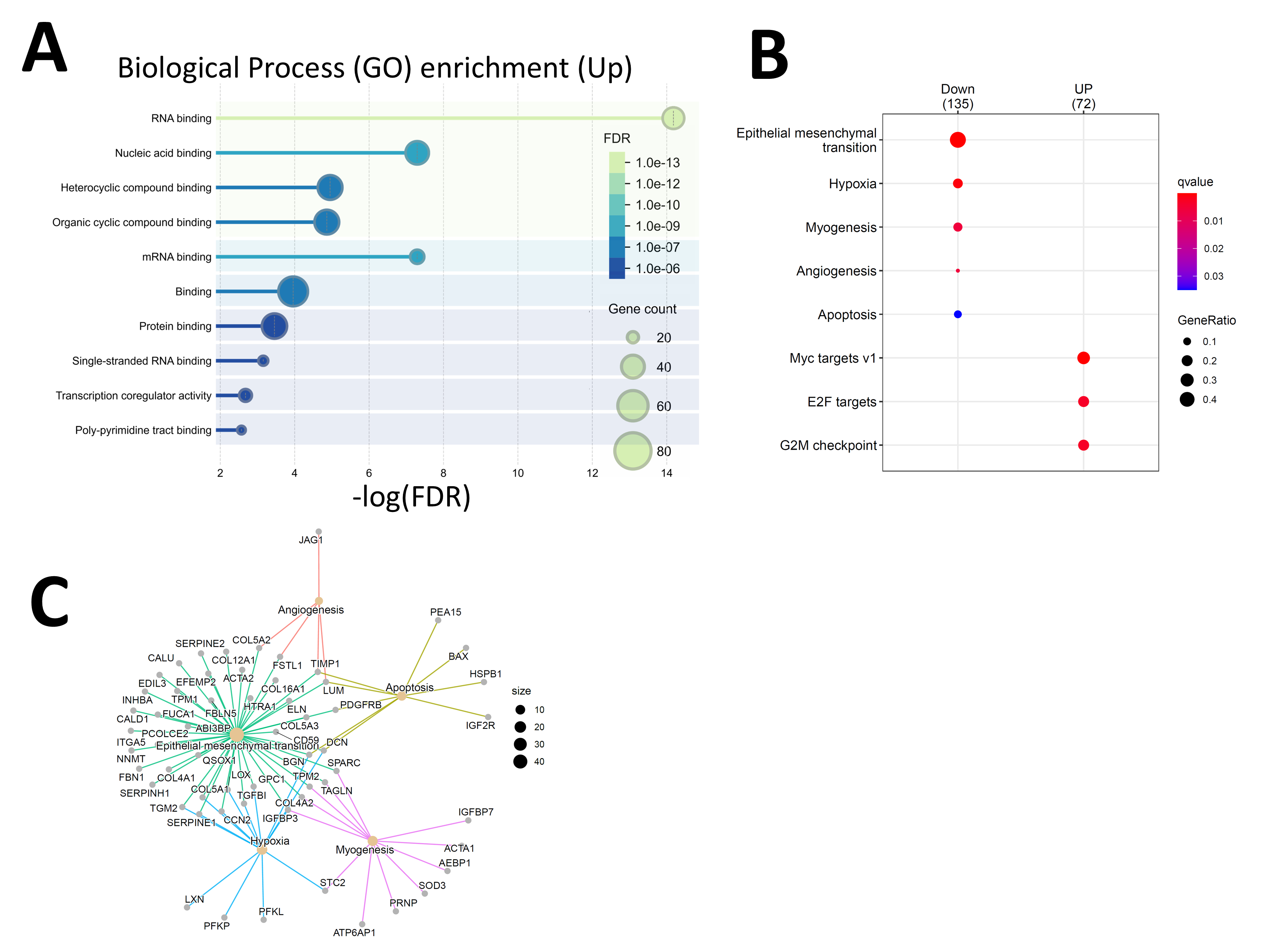

Supplement: Supplementary file 2 — Supplementary Material 2: Supplementary Figure. (A) Biological process Gene Ontology (GO) enrichment analysis revealed that RNA binding was significantly upregulated in the f-hPL group. (B) Protein degree analysis revealed that stemness marker (Myc targets v1) were upregulated in f-hPL group, while epithelial mesenchymal transition was downregulated. (C) Protein-protein interaction network analysis of downregulated proteins. [file 13287_2025_4329_MOESM2_ESM.tif]
