# Supplementary material for: Human platelet lysate produced from leukoreduction filter contents enables sufficient MSC growth
Source: Stem Cell Res Ther. 2025 Apr 23;16:205. doi: 10.1186/s13287-025-04329-y (PMC12020118; doi:10.1186/s13287-025-04329-y)
Supplement: Supplementary file 3 — Supplementary Material 3 [file 13287_2025_4329_MOESM3_ESM.docx]

Infectious testing of leukoreduction filter

All filters used in this experiment are provided by the Japan Red Cross Association, the only blood supplier in Japan. These filters are employed after meeting the rigorous criteria applied to all blood products intended for patient use. The criteria include: 1) completion of a health check self-assessment, 2) successfully undergoing antigen/antibody testing for syphilis antibody, HBs antigen, HBc antibody, HBs antibody, HCV antibody, HIV antibody, HTLV-1 antibody, and PV-B19 antigen, and 3) passing nucleic acid amplification testing (NAT) for HBV, HCV, HIV, and HEV.

SWATH‑MS‑Based Quantitative Proteomics

Samples were digested using the phase-transfer surfactant method as previously described.(1, 2) A TripleTOF 5600 mass spectrometer (Sciex, Framingham, MA, USA) coupled with the Dionex Ultimate 3000 RSLCnano System (Dionex, Sunnyvale, CA, USA) was used for SWATH-MS. The peptides were filtered at a false discovery rate of < 1% for identification and quantification. Proteins were identified using ProteinPilot v.4.5 (Sciex) using mass spectroscopy data from information-dependent acquisition and UniProt rat reference proteome data. DIA-NN v.1.7 (3) was utilized to analyze the peptide peaks from SWATH data using a spectral library constructed using the identification data. The MaxLFQ algorithm was used to calculate the protein expression levels from the precursor peak areas (3); the concentration of each protein was calculated as the peak area of the protein obtained by summing the peak areas of all the specific tryptic peptides. Differential expression protein analysis was performed using RNAseqChef (https://imeg-ku.shinyapps.io/RNAseqChef_imeg/).(4) Pairwise differential expression analysis between the Pre-signal group and the Signal group was conducted using the limma package for normalized count data, as implemented in RNAseqChef. Given the exploratory nature of this study, proteins with false discovery rate (FDR) less than 0.1 were considered as significantly differentially expressed, if those proteins also had a fold change of lower/higher than -1.2/1.2, they were defined as being down-/upregulated, respectively. Network and Gene Ontology (GO) analysis of the differentially expressed proteins was conducted using STRING version 12.0 (https://string-db.org/).(5) Protein degree analysis was performed using Cytoscape software (Version 3.10.3) to identify key proteins within the network based on their connectivity and influence.(6)

1. Masuda T, Tomita M, Ishihama Y. Phase transfer surfactant-aided trypsin digestion for membrane proteome analysis. J Proteome Res. 2008;7(2):731-40.

2. Mori A, Masuda T, Ito S, Ohtsuki S. Human Hepatic Transporter Signature Peptides for Quantitative Targeted Absolute Proteomics: Selection, Digestion Efficiency, and Peptide Stability. Pharm Res. 2022;39(11):2965-78.

3. Demichev V, Messner CB, Vernardis SI, Lilley KS, Ralser M. DIA-NN: neural networks and interference correction enable deep proteome coverage in high throughput. Nat Methods. 2020;17(1):41-4.

4. Etoh K, Nakao M. A web-based integrative transcriptome analysis, RNAseqChef, uncovers the cell/tissue type-dependent action of sulforaphane. J Biol Chem. 2023;299(6):104810.

5. Szklarczyk D, Gable AL, Nastou KC, Lyon D, Kirsch R, Pyysalo S, et al. The STRING database in 2021: customizable protein-protein networks, and functional characterization of user-uploaded gene/measurement sets. Nucleic Acids Res. 2021;49(D1):D605-D12.

6. Shannon P, Markiel A, Ozier O, Baliga NS, Wang JT, Ramage D, et al. Cytoscape: a software environment for integrated models of biomolecular interaction networks. Genome Res. 2003;13(11):2498-504.
